# Evaluating the Influence of Flaxseed Oil Against Docosahexaenoic Acid on Cancer Antigen CA15‐3, Biochemical and Histopathological Parameters of DMBA‐Induced Mammary Tumors Female Albino Wistar Rats

**DOI:** 10.1002/fsn3.71969

**Published:** 2026-06-03

**Authors:** Hilda Nji Aza, Fabrice Tonfack Djikeng, Achidi Aduni Ufuan, Ines Sorelle Fotsing, Stephane Zingue

**Affiliations:** ^1^ School of Home Economics, Tourism and Hotel Management, Chartered Higher University Institute of Technology and Management Buea Cameroon; ^2^ Department of Biochemistry and Molecular Biology, Faculty of Science University of Buea Buea Cameroon; ^3^ Basic and Clinical Cancer Research Unit (BCCRU), Department of Pharmacotoxicology and Pharmacokinetics, Faculty of Medicine and Biomedical Sciences University of Yaoundé 1 Yaoundé Cameroon

**Keywords:** biochemical and histopathology markers, breast cancer, cancer antigen CA15‐3, DHA, flaxseed oil

## Abstract

The effect of flaxseed oil on the biochemical, CA15‐3 markers and organs' microstructures of female rats with breast cancer induced using the 7,12‐Dimethylbenz[a]anthracene (DMBA) against docosahexaenoic acid (DHA) was assessed. 36 female rats were used. Breast cancer was induced in 30 rats using the DMBA. At week 19, a total of 23 rats had developed tumors. Amongst them, 18 were selected and assembled in three groups of six rats each: The negative control to which was administered 250 mg/kg of distilled water, the positive control that received 125 mg/kg of DHA, the test group which was given 1000 mg/kg flaxseed oil and the normal group made of rats that were not induced but received 250 mg/kg of distilled water for 28 consecutive days. The rats were sacrificed and the blood collected to prepare the serum that was analyzed for the lipid profile, serum enzymes, breast cancer tumor marker, and creatinine levels. Histopathology of organs was done. Results showed that flaxseed oil and DHA increased the HDL and decreased the LDL, CA15‐3, LDH, creatinine, AST and ALT compared to the negative control. Histopathological studies revealed protective properties of DHA and flaxseed oil on the organs.

## Graphical abstract

Flaxseed oil and docosahexaenoic acid administered to female Wistar rats with DMBA‐induced breast cancer help increase the HDL cholesterol and significantly reduce the CA15‐3, AST, ALT, LDL, and creatinine levels of rats compared to the negative control group. They also exhibited a protective effect on organs from histopathological data.

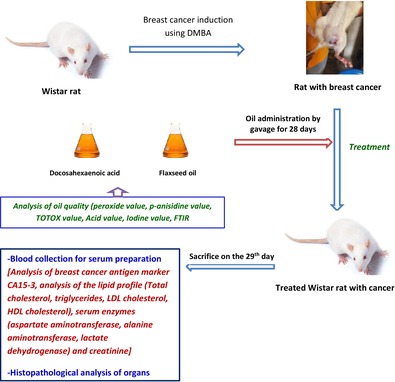

## 1 Introduction

Mammary cancer is known as the most diagnosed cancer in the world (IARC 2025) and represents the leading major health challenge, displaying important regional disparities in incidence, survival rates and mortality (Freihat et al. 2025). Its rate continues to increase in a steady manner with about 2.3 million of new cases diagnosed globally every year (Łukasiewicz et al. 2021). The risk factors associated to breast cancer include environmental factors such as pollutants (polycyclic aromatic hydrocarbons), changes in lifestyle, smoke etc., age, genetic factors etc. (Sung et al. 2017; Zingue et al. 2024). Reports show that without intervention, global cancer cases are expected to rise by 49%, with mortalities mounting by 62% by 2040 (Cao et al. 2021). In Africa, mammary cancer is the top reason of malignancy deaths in women, representing about 28% of all female cancers and 20% of cancer‐related demises (Ferlay et al. 2020). In Cameroon, it continues to be a significant challenge, with over 4170 new cases (20.1%) and 2108 deaths (16%) each year (Sung et al. 2021; Zingue et al. 2021). Women at a specific age are recommended to go for regular screening for early detection and those who are at high risk can start taking preventive treatment which can include preventive therapy with tamoxifen or surgical prophylaxis (Sahin et al. 2011).

In order to address the challenges faced by the burden cancer, options such as surgery, radiation therapy, immunotherapy, chemotherapy, cancer drugs etc. have been used. However, due to their high cost, they are not accessible to everyone and have been proven as leading to several side effects (Vanderpuye et al. 2017). Alternatively, natural substances are gradually gaining a lot of interest in fighting cancer, especially in middle‐ and low revenue developing nations where about 80% of the people seek for natural products instead of synthetic ones because they are considered to be safer and cheaper (Zingue et al. 2024; Khazaei et al. 2015). Amongst the natural products are functional food ingredients and nutraceuticals. It is believed that diet rich in nutraceuticals or functional food ingredients as well as food supplements can help in avoiding and managing a wide range of ailments amongst which cancer. Amongst them, omega‐3 (ω3) fatty acids or food sources containing them are highly solicited in the prevention and management of cancer. Research indicates that ω3 fatty acids inhibits the proliferation of breast cancer cells induced apoptosis, potentially reduces tumor growth etc. (Theinel et al. 2023). The ω3 fatty acids that have been highly related to anti‐cancer activities are docosahexaenoic acid and eicosapentaenoic acid from fish oil (Du et al. 2019; West et al. 2020). The influence of eicosapentaenoic acid, docosahexaenoic acid and fish oil on mammary cancer in female rats induced with DMBA has been intensively reported (Chatterjee et al. 2010; Manna et al. 2011; Siddiqui et al. 2013; Noguchi et al. 1997). In other studies, alpha‐linolenic acid, an omega‐3 fatty acid from animal or plant origin was also reported to be efficient in DMBA‐induced mammary gland carcinoma (Tripathi et al. 2018). It is important to know that most of the oils consumed in the world are from plant origin (Vegetable oils). The omega‐3 fatty acid found in them is α‐linolenic acid. It will be important to discover the potential impact of the consumption of oils from plant origin rich in omega‐3 fatty acids (α‐linolenic acid) on the prevention and management of some cancers such as breast cancer. In one study, Zingue et al. (2024) highlighted the effectiveness of Njansang seed oil, a vegetable oil rich in omega‐3 fatty acids in the prevention of breast cancer in menopause‐like conditions female wistar rats with malignant tumor of the breast induced using DMBA. Most of the other studies involving vegetable oils have been done in vitro against cancer cells (Batool et al. 2023; Gazem et al. 2017; Merichi et al. 2017; Al‐Hwaiti et al. 2020). There is a need to explore additional sources of omega‐3 fatty acids from plant origin that after consumption can help in the prevention or management of breast cancer induced with the DMBA in contrast with DHA, a proven ω3 fatty acid with demonstrated anti‐cancer property. Amongst the oilseed rich in ω3 fatty acids is flaxseed, which is highly sold and used for consumption in Cameroon but minimally produced. Flaxseed market in Cameroon is primarily driven by imports of seeds and oil rather than large‐scale domestic cultivation (6Wresearch 2026).

Flax ( *Linum usitatissimum* ) belongs to the Lineaceae family. Its seeds are reddish brown to golden yellow in color. They possess a nutty taste and are crispy in texture (Rubilar et al. 2010). Flaxseed is known for its potential health welfares due to its high concentration of ω3 linolenic acid, roughage and lignins. Polyunsaturated fatty acids from flaxseed might help against diseases such as cancer, cardiovascular diseases, atherosclerosis, hypertension, diabetes, etc. (Gogus and Smith 2010; Simopoulos 2000). Flaxseed oil contains monounsaturated, saturated and polyunsaturated fractions amongst which the unsaturated fatty acids represent about 87.8%–89.8% (Yacoob and Hassan 2016). The major constituents of flaxseed oil are linoleic acid (26.2%), α‐linolenic acid (42.5%), stearic acid (10.7%), and palmitic acid (12.9%) (Ishag et al. 2020). The effect of this oil on breast cancer induced in female rats using DMBA has not yet been reported despite its health benefits (Akrami et al. 2018). However, it was demonstrated to be efficient in killing cancer cells (Batool et al. 2023). This study aimed to appraise the influence of flaxseed oil on some biochemical and histopathological parameters of female Wistar rats with breast cancer induced using DMBA against docosahexaenoic acid. Wistar rats were chosen because they are known as foundational laboratory animal specimens due to their physiology, genetics and metabolism that are similar to human biology (Patel et al. 2024).

## 2 Materials and Methods

### 2.1 Materials

Docosahexaenoic acid powder and the cancer inducer [7, 12‐dimethylbenz (a) anthracene and abbreviated DMBA] were bought from sigma‐Aldrich (Starnberg, Germany). The other chemicals and solvents used in this study were of good quality. Flaxseed, the reddish brown variety, in its dried form was purchased from a shop in the town of Douala, Littoral Region, Cameroon in November 2024. The seeds originated from the Himachal Pradesh region, India. Reports show that in this agricultural region of India, flaxseed grows best in well‐drained sandy loam to silty clay loam soils, with optimal cultivation in soils with pH ranged between 5.5–7.0. The climate adequate for flaxseed production in this region is sub‐tropical, and the conditions for optimal growth being as follows: Temperature, 10°C–27°C, humidity, 60%–65%, and annual rainfall 155–200 mm (Singh and Chopra 2018). Female rats (36), belonging to the Wistar strain and aged 6–9 weeks (55–60 g) were acquired from a farmer in Yaoundé, Center Region, Cameroon in September 2024.

### 2.2 Methods

#### 2.2.1 Extraction and Quality Analysis of Flaxseed Oil

The technique described by Bligh and Dyer was used for the extraction of flaxseed oil (Bligh and Dyer 1959). Flaxseed was ground and 100 g of it was introduced into a blender, followed by the addition of methanol (200 mL). The mixture was homogenized for about 2 min and chloroform (100 mL) was added. After homogenizing again for 2 min, another 100 mL of chloroform and 100 mL of distilled water was added. The mixture was homogenized again for 2 min and then filtrated using a muselin cloth. The cake was discarded and the filtrate re‐filtered again using a most selective filter, the Whatman paper (no. 1). The new filtrate was introduced into a separation funnel and allowed for 24 h. After that, the phases containing water and methanol were discarded. The one containing chloroform and oil was collected and evaporated under vacuum on a rotatory evaporator at to eliminate the solvent. The extracted oil was packaged and conserved in the freezer (−18°C) for future use. The oil was later on analyzed for its quality. The peroxide value (PV) was assessed using the protocol of the IDF 74A: 1991 (IDF 1991). The p‐Anisidine (p‐AnV) and the acid (AV) values were measured using the AOCS Official Protocols Cd 18–90 and Cd 1–15 (AOCS 2003) respectively. The iodine value (IV) was determined using the AOCS protocol Cd 1–25 (AOCS 2003). The thiobarbituric acid value (TBA) was determined as reported by Draper and Hadley (1990). The oil quality was also analyzed using Fourier‐transformed infrared spectroscopy (FTIR) as described by Liang et al. (2013).

#### 2.2.2 Animal Bioassay

##### 2.2.2.1 Ethical Clearance

The rats used in this study were treated according to the international standard guideline for animal use. A clearance for ethics (reference number UB‐ IACUC No. 02/2025) was gotten from the Institutional Animal Care and Use Committee, University of Buea.

##### 2.2.2.2 Animal Feed Composition

The standard diet was composed as reported by Zingue et al. (2024). The ingredients and their proportions are presented in Table 1.

**TABLE 1:** Feed composition and proportion.

S/N	Ingredient	Proportion (%)
1	Wheat meal	36.6
2	Corn‐meal	36.7
3	Crushed fish	4.8
4	Bone meal	14.5
5	Iodide salt	0.3
6	Palm kernel cake	7.3
7	Vitamin	0.01

##### 2.2.2.3 Animal Randomization and Feeding

36 female wistar rats aged 6–9 weeks (55–60 g) were used in work. Upon arrival of the rats, they were permitted to adapt for 7 days under a 12‐h light–dark cycle. Their cages contained sawdust and they had free access to food and water. Out of the 36 rats, 30 were anesthetized by intraperitoneal administration of diazepam (10 mg/kg) and ketamine (50 mg/kg). Breast cancer was induced in them by injecting a unique dose (50 mg/kg) of DMBA liquefied in 1 mL olive oil via subcutaneous intra‐mammary route. The remaining 6 animals were considered as normal control. In view to prevent any type of infection that can lead to the animal sickness or death, the rats in which DMBA was injected and those of the normal group were given amoxicillin (89 mg/kg) by gavage for 10 successive days (Zingue et al. 2024). Tumor development was checked after every two days through palpation. Generally, tumors started showing around week 19. Out of the 30 rats induced for breast cancer, 23 rats developed tumors. 18 of them were selected according to their tumor sizes and distributed into three groups of six rats each. The 6 rats that were not induced for cancer (normal group) represented the fourth group, for a total of 24 rats. Throughout the period of tumor development, wounds were appearing at the induction site. They were treated using betadine and penicillin ointment twice daily. They were randomized as presented in Table 2 which includes: the normal group (Group 1) which was given distilled water (250 mg/kg) by gavage for 28 days, the negative control group (Group 2) which was not treated and was given distilled water (250 mg/kg) daily by gavage for 28 days, the positive control group (Group 3) which was given docosahexaenoic acid daily at the concentration 125 mg/kg (El‐Mesery et al. 2009) by gavage for 28 days, and the test group (Group 4) which was given flaxseed oil daily at the concentration 1000 mg/kg (Zingue et al. 2024; Djikeng et al. 2025).

**TABLE 2:** Animal group distribution.

Groups	
Group 1 (normal)	Normal rats receiving distilled water (250 mg/kg)
Group 2 (negative control)	Rats with cancer receiving distilled water (250 mg/kg) by gavage for 28 days
Group 3 (positive control)	Rats with cancer receiving Docosahexaenoic acid daily at the dose 125 mg/kg by gavage for 28 days
Group 4 (test group)	Rats with cancer receiving oral administration of 1000 mg/kg of flaxseed oil daily by gavage for 28 days

##### 2.2.2.4 Measurement of Body Weight

The method described by Ru et al. (2018) was used in this project. An electrical scale was used to monitor the body weight throughout the experimental period. The body weight was taken at 7 days intervals till the end of the experimental period.

##### 2.2.2.5 Animal Sacrifice and Collection of Samples

After 28 days of treatment, the animals were allowed to fast overnight. They were sacrificed on the 29th day. Before the sacrifice, the animals were sedated through intraperitoneal injection of Diazepam and Ketamine (10 and 60 mg/kg respectively). The anesthetized rat models were then sacrificed by cutting their abdominal artery. Blood was collected by cardiac puncture using a syringe of 5 mL volume. The blood was transferred in dry tubes (one with anticoagulant and one without anticoagulant). Blood samples in tubes without anticoagulants were centrifuged for 15 min at 3500 rpm to obtain the serum. The serum was kept at −18°C for further characterizations. Organs of interest were weighed (heart, kidney, pancreas, brain, and liver) and used for histopathological studies.

##### 2.2.2.6 Exploration of Biochemical Parameters

These parameters included the measurement of serum enzymes [lactate dehydrogenase (LDH), alanine transferase (ALT), aspartate transferase (AST)], the lipid profile (triglycerides, total cholesterol, LDL cholesterol, HDL cholesterol), and the kidney function marker (creatinine) following the protocol described in the commercial kits SGM notice.

##### 2.2.2.7 Measurement of Breast Cancer Tumor Marker

ELISA (sandwich enzyme‐linked immunosorbent assay) was used for the measurement of breast tumor marker. The Biovantion Liusan CA 15‐3 ELISA kit protocol was used to measure the cancer antigen CA15‐3 concentration in rat serum.

##### 2.2.2.8 Histopathology of Organs

The potential pathologies and microscopic structure of organs were examined as described by Cardiff et al. (2014). Small sections of breast tumor, kidneys, liver, brain, heart, and pancreas were collected and treated for detailed cellular microscopic assessment. Tissue pieces were inspected using a light microscope coupled to a computer. ×100 and ×200 magnifications were used to assess the morphological features of organ sections. The cellular architecture and pathological changes were evaluated, comprising inflammation, marked by characteristics of fibrosis, infiltration of leukocytes, and tumor.

##### 2.2.2.9 Statistical Analysis

Oil characterization was performed in triplicate (*n* = 3) while animal bioassays were done sixfold (*n* = 6). Data were submitted to one‐way analysis of variance (ANOVA). The Student–Newman–Keuls test was used to assess the statistical significance of the data using the software Statgraphics Centurion version XVI. Statistical significance was considered at probability *p* < 0.05.

## 3 Results

### 3.1 Oil Quality Markers

**FIGURE 1:**
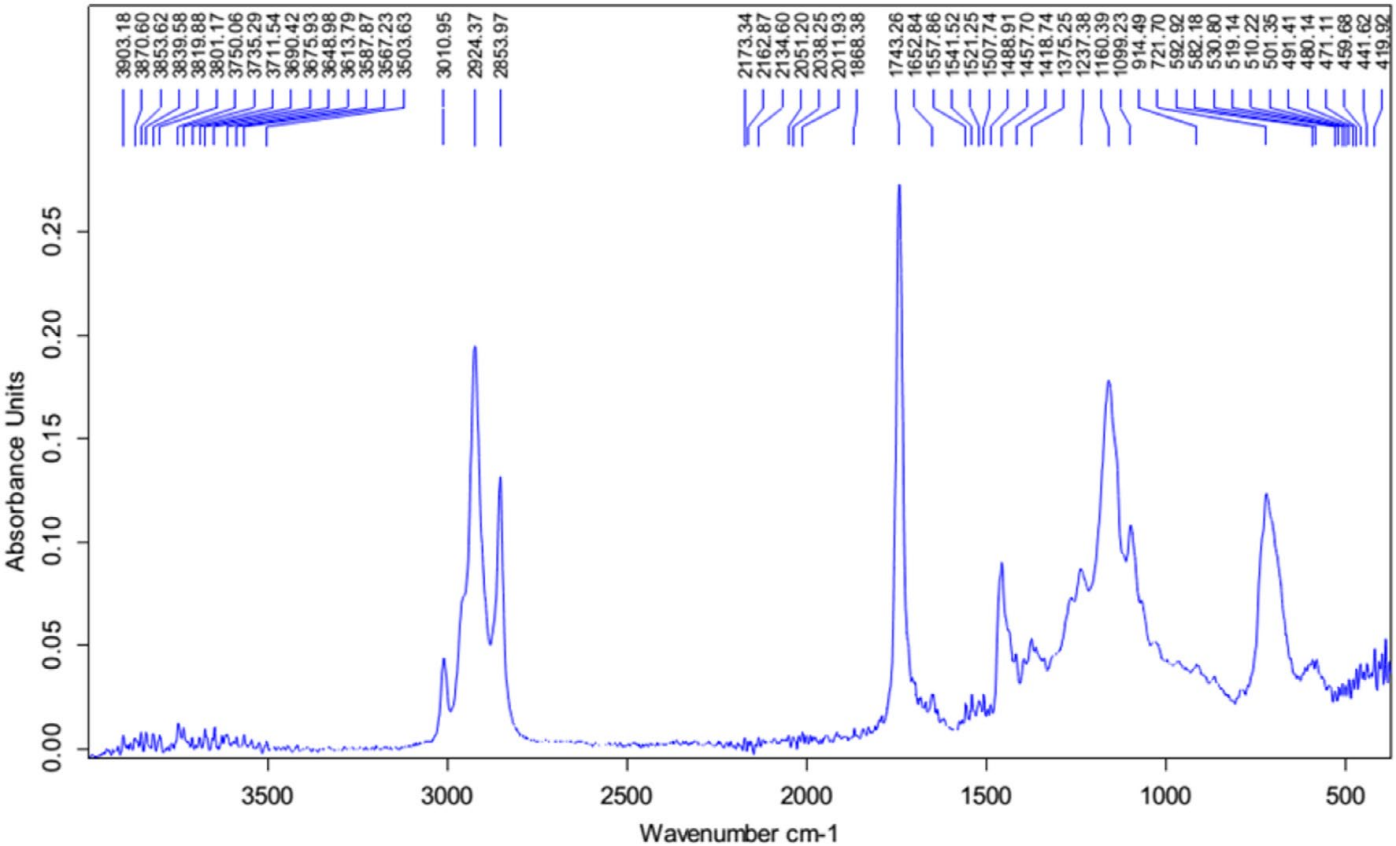
Fourier‐transformed infrared spectrum of flaxseed oil.

**FIGURE 2:**
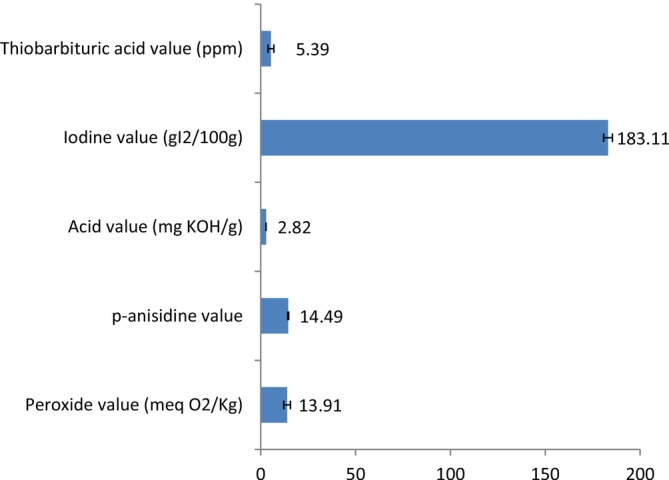
Quality indexes of flaxseed oil.

Figure 1 presents the FTIR spectrum of flaxseed oil. Peaks of high intensity were noted around 2921, 2854, 1743, 1500, 1160, and 721 cm^−1^. Some peaks were also observed around 3750, 3010, and 1650 cm^−1^ but with lower intensities. The quality indexes (Figure 2) of flaxseed oil were respectively 13.91 meq O_2_/kg, 14.49, 2.82 mg KOH/g, 183.11 g *I*_2_/100 g and 5.39 ppm for the PV, p‐AnV, AV, IV and TBAV values.

### 3.2 Body and Organs Weights

**FIGURE 3:**
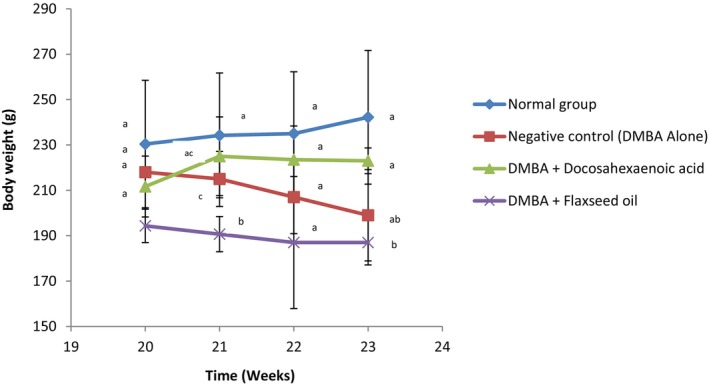
Variation in body weight of Wistar rats throughout the experiment. *n* = 6. Values are presented as mean ± standard deviation. ^a‐c^Values with different superscripts are significantly different at *p* < 0.05.

Figure 3, presents the changes in body weight of animals throughout the experiment. A significant (*p* < 0.05) decrease in body weight was observed in the group taking flaxseed oil (3.61%) and the negative control group (8.72%) while the weight of rats from the normal group and the group receiving DHA increased by 5.12% and 5.69% respectively.

**FIGURE 4:**
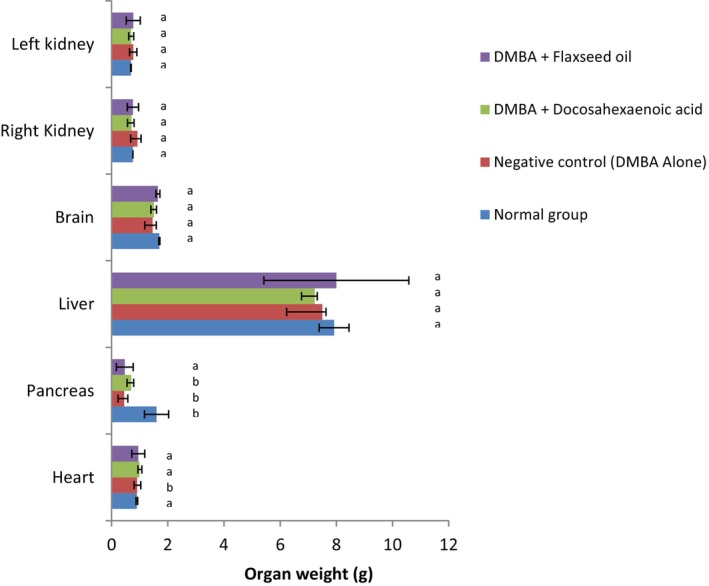
Weight of organs. *n* = 6 values are presented as mean ± standard deviation. ^a,b^Values of the same column with different superscript are significantly different at *p* < 0.05. Normal: Rats without cancer that received distilled water; DMBA + Docosahexaenoic acid: Rats with breast cancer treated with docosahexaenoic; Negative control: Rats with breast cancer and untreated; DMBA + Flaxseed oil: Rats with breast cancer treated with flaxseed oil.

The organs' weight of the rats after sacrifice is exhibited in Figure 4. No difference was recorded in the weight of the right kidney, heart, left kidney, liver, and brain across all groups. The highest (*p* < 0.05) weight for the pancreas was observed in the normal group (1.60 g) while this parameter was significantly lower (*p* < 0.05) in DMBA‐treated groups (0.45–0.70 g).

### 3.3 Lipid Profile

**FIGURE 5:**
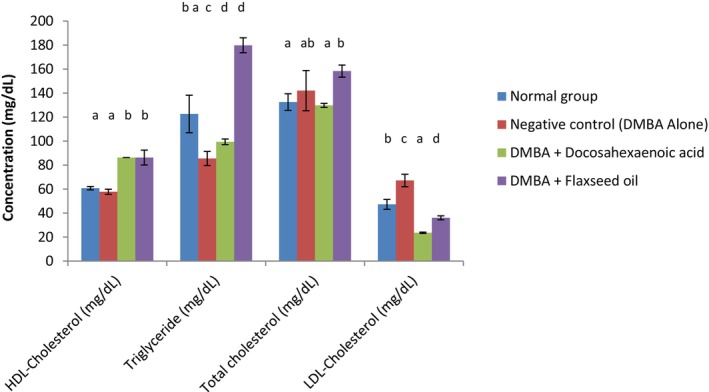
Lipid profile of DMBA‐induced breast cancer Wistar rats. Normal: Rats without cancer that received distilled water; DMBA + Docosahexaenoic acid: Rats with breast cancer treated with docosahexaenoic acid; Negative control: Rats with breast cancer and untreated; DMBA + Flaxseed oil: Rats with breast cancer treated with flaxseed oil. *n* = 6. Values are presented as mean ± standard deviation. ^a–d^values of the same column with different superscripts are significantly different at *p* < 0.05.

Figure 5 presents the lipid profile of normal and DMBA‐induced mammary tumor in female rats. The negative control group showed the lowest (*p* < 0.05) HDL concentration (57.75 mg/dL). The groups taking DHA and flaxseed oil exhibited higher (*p* < 0.05) HDL cholesterol (86.29 mg/dL). Looking at the triglyceride, the negative control group also presented the lowest (*p* < 0.05) concentration (85.50 mg/dL) compared to the other groups. The group taking flaxseed oil and the normal group showed significantly (*p* < 0.05) higher triglyceride levels (122.60 and 179.88 mg/dL respectively). The total cholesterol was considerably (*p* < 0.05) higher in the group fed with flaxseed oil (158.33 mg/dL) compared to the others (129.76–142.06 mg/dL). Higher LDL cholesterol was noted in the negative control group (67.20 mg/dL).

### 3.4 Serum Enzymes, CA15‐3 Tumor Marker and Creatinine Level

**FIGURE 6:**
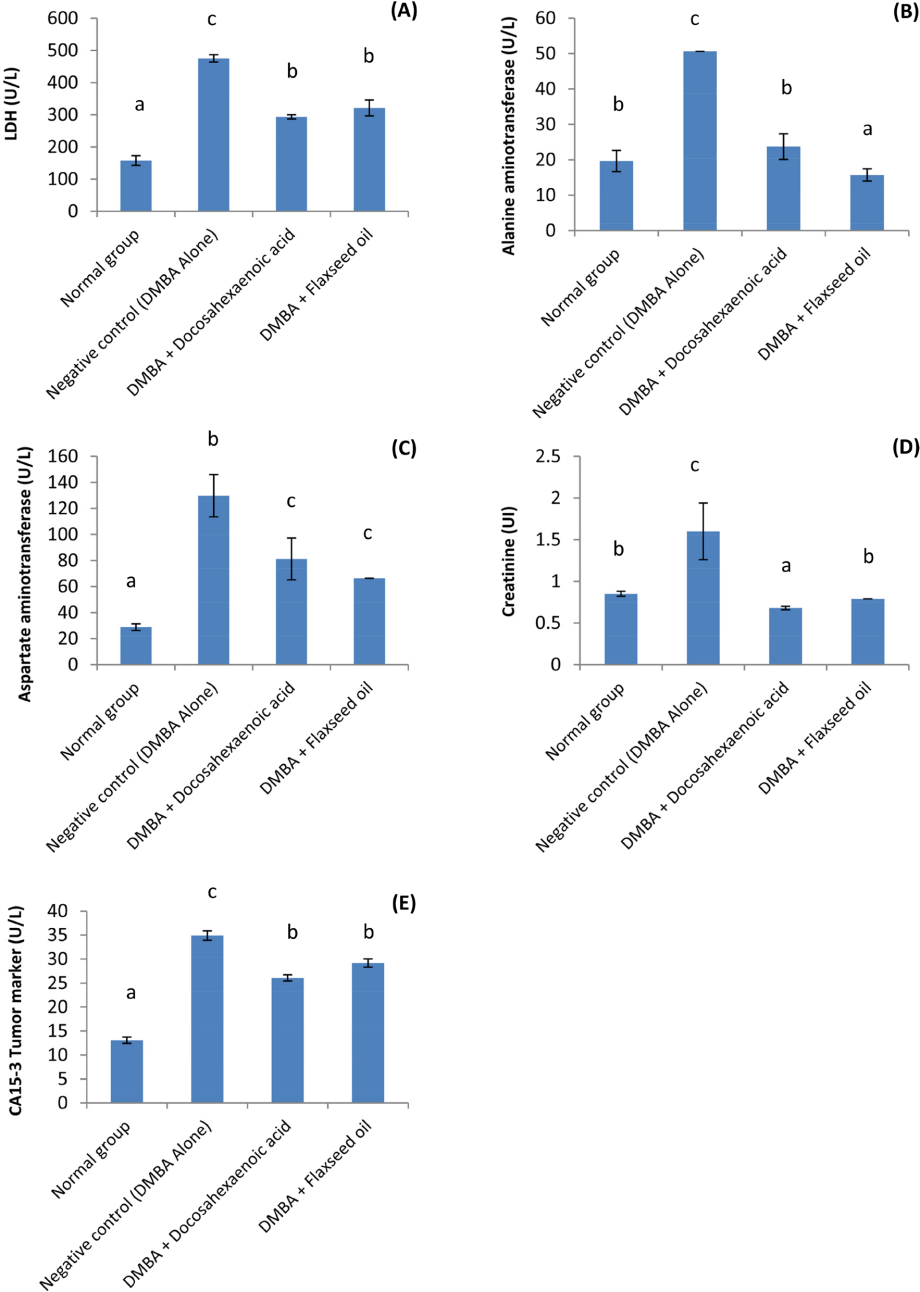
(A–E) LDH (lactate dehydrogenase) (A), ALT (Alanine aminotransferase) (B), AST (Aspartate amino transferase) (C), Creatinine (D) and CA15‐3 tumor marker (E) levels of DMBA‐Induced Breast cancer Wistar rats. Standard deviation. Normal: Rats without cancer that received distilled water; DMBA + Docosahexaenoic acid: Rats with breast cancer treated with docosahexaenoic; Negative control: Rats with breast cancer and untreated; DMBA + Flaxseed oil: Rats with breast cancer treated with Flaxseed oil. *n* = 6. Values are presented as mean ± standard deviation. ^a–c^values of the same column with different superscripts are significantly different at *p* < 0.05.

The serum enzymes, creatinine level and CA‐15‐3 tumor marker concentrations of animals are presented in Figure 6A–E. The negative control group showed significantly (*p* < 0.001) higher creatinine (1.6 UI), CA‐15‐3 (34.91 U/L), LDH (475.31 U/L), AST (129.78 U/L) and ALT (50.63 U/L) levels compared to the other groups (0.68–0.85 UI, 13.07–29.16 U/L, 157.85–321.27 U/L, 28.81–66.35 U/L, 15.71–23.74 U/L respectively).

### 3.5 Histopathological Studies

**FIGURE 7:**
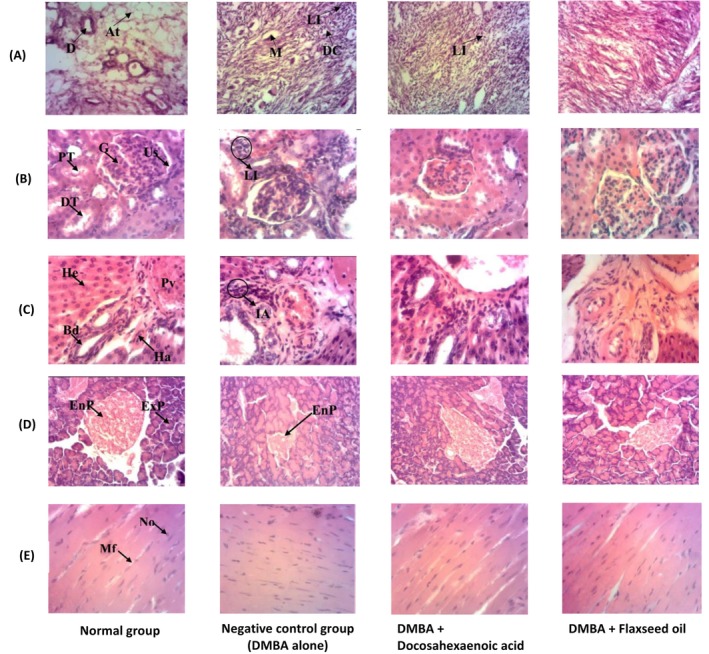
(A–E): Effects of docosahexaenoic acid and flaxseed oil microarchitectures of breast tissues (A), kidney (B), liver (C), pancreas (D), and heart (E). At, adipocytes tissue; Bd, bile duct; D, ductal lumen; DC, ductal carcinoma; DT, distal tube; EnP, endocrine part of the pancreas; ExP, exocrine part of the pancreas; G, glomerulus; Ha, hepatic artery; He, hepatocytes; IA, inflammatory area; Li, leucocyte infiltrations; M, mucinous secretions; Mf, muscle fiber, No, nucleus; Pv, portal vein; Us, urinary space. Normal: Rats without cancer that received distilled water; DMBA + Docosahexaenoic acid: Rats with breast cancer treated with docosahexaenoic; Negative control: Rats with breast cancer and untreated; DMBA + Flaxseed oil: Rats with breast cancer treated with flaxseed oil.

The histopathology of the tumor and the other organs is presented in Figure 7A–E.

The images of the breast tissues (tumor) are presented in Figure 7A. The breast tissue of the negative control highlights the presence of mucin in the ductal lumen and granules in the cytoplasm. Discontinuity is observed in some ducts of the basement membrane with papillary outgrowth of malignant cells. The degree of differentiation was variable and suggestive of adenocarcinoma due to the presence of neoplastic cells organized in acinar and tubular formations. Streaming patterns and pleomorphism were also observed. The tumor cells were covered with a thick mass of fibrous connective tissue. Flaxseed and DHA corrected this alteration.

Figure 7B shows pictures of kidney sections from different groups. Rats of the normal group exhibited normal kidney structure. The negative control group outlined mesangial expansion and leukocyte infiltrations. Administration of flaxseed oil and docosahexaenoic acid prevented the kidney from alterations.

Concerning the liver (Figure 7C), its structure remained unaltered in rats from the normal group. However, in the negative control, the infiltration of leukocytes is noted in the portal space, reflecting an inflammatory reaction. Gavaging animals with flaxseed oil and docosahexaenoic acid led to the total disappearance of this inflammation zone.

The histopathology of the pancreas is exhibited in Figure 7D. The microphotograph of a normal pancreas shows a normal architecture composed of an endocrine section (EnP) containing the islets of Langerhans and an exocrine part (ExP) with acinar cells. In the negative control, we observe a reduction in the size of the pancreatic islets compared to the normal control. This reflects the decrease in secretory activity. The photograph of the pancreas of the positive control as well as those that received flaxseed oil and DHA shows a slightly altered architecture with islets with irregular contours.

Looking at the heart (Figure 7E), in normal subjects, cardiac parenchyma is noted to have elongated and tapered muscular fibers; each containing numerous nuclei. In negative subjects, nuclei of cardiac cells are disorganized compared to the normal control.

**FIGURE 8:**
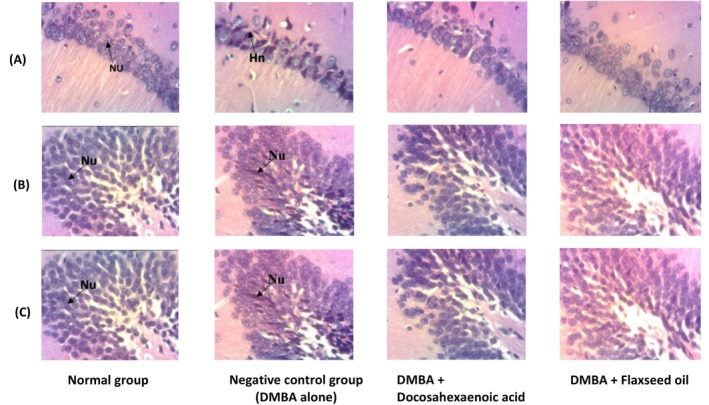
(A–C) Effects of docosahexaenoic acid and flaxseed oil microarchitectures of CA1 area of hippocampus (A), CA3 area of hippocampus (B), and dentate gyrus of hippocampus (C). Nu: Nucleus, Hn: Hyperchromatic nuclei. Normal: Rats without cancer that received distilled water; DMBA + Docosahexaenoic acid: Rats with breast cancer treated with docosahexaenoic acid; Negative control: Rats with breast cancer and untreated; DMBA + Flaxseed oil: Rats with breast cancer treated with flaxseed oil.

Figure 8A–C also shows the effects of flaxseed oil and DHA on the microstructure of the horns of Amun 1 and 3 (CA1 and CA3) and the dentate gyrus of the hippocampus. Results show that administration of 7,12‐Dimethylbenz[a]anthracene leads to the formation of nuclei (hyperchromatic) in the CA1 region of the hippocampus (Figure 8A), reflecting the death of brain cells. Administration of docosahexaenoic acid and flaxseed oil protected and preserved the integrity of neuronal nuclei (absence of hyperchromatic nuclei) in the CA1 region of the hippocampus. In the CA3 region (Figure 8B), no difference was noted among the group taking flaxseed oil, the normal group, and the negative control. At the level of the dentate gyrus (Figure 8C), hyperchromatic nuclei were observed in abundance in the negative control.

## 4 Discussion

Oils and fats are of great importance in our diet. However, it is always better to have an idea on their quality for safety reasons, and to understand their functionality. Quality indexes and FTIR are some of the techniques generally used to test oil quality, especially their oxidative state. In this light, FTIR provides information about the presence or not of specific functional groups characteristic of oxidation reactions. Quality indexes, on the other hand, give additional insight on the primary, secondary, and hydrolytic oxidation state of oils, as well as their degree of unsaturation. The analysis of oil quality showed peaks with low intensity around 3750 cm^−1^. Reports show that peaks observed in that area generally characterize the O‐H stretching vibration of molecules such as alcohol, water, or hydroperoxides. The low intensity of these peaks might be a reflection of low primary oxidation, as reported by Shi et al. (2017). The peaks registered on the FTIR spectrum around 3000 and 2921 cm^−1^ match with the stretching vibrations of carbons in the structure of alkenes (hybrid) and alkanes respectively (Coates et al. 2000). The peak at 3000 cm^−1^ can be linked to the double bonds found in the aliphatic chain of polyunsaturated fatty acids (Coates et al. 2000). The important peak at 1743 cm^−1^ is generally associated with the stretching vibration of C=O functional groups which are found in oxidation products such as ketones, aldehydes, and esters (Douky et al. 2025). The FTIR spectrum exhibited no peak at 2750 and 1600 cm^−1^. These areas are generally assigned to the C—H of aldehydes and C=C of aromatic rings and can attest to very low or no secondary oxidation of the lipids in the oil (Coates et al. 2000). Lastly, important peaks were recorded around 721 and 1000 cm^−1^. They were respectively reported to match with the out‐of‐plane bending vibration of C—H bonds present in the structure of alkenes or aromatic molecules and the C—H of aromatic molecules (Silverstein et al. 2005). Globally, the above‐mentioned information shows that the flaxseed oil used was of acceptable quality (Douky et al. 2025).

Data from the analysis of quality indexes revealed peroxide value of 13.91 meq O_2_/kg. In is important to known that this oil quality marker provides insights on the amount of hydroperoxides released in the oil, which is the consequence of their primary oxidation. The value obtained for flaxseed oil was < 15 meq O_2_/kg which is the approved range as per the standards (FAO/WHO 1999). In the same line, the p‐anisidine value of this oil was found to be 14.49 which is far lower than 20 as recommended by the norm in fish oil (FAO/WHO 2017). Since the standard for this oxidation marker is not available for oils from plant origin, we used the one of fish oil to conclude on the quality of the flaxseed oil used. The p‐anisidine value quantifies 2‐alkenals and 2, 4‐dienals which are aldehydes produced in the oil undergoing secondary alteration. Another parameter used to evaluate this type of oil spoilage by qualifying similar products is the thiobarbituric acid (TBA) value. The aldehyde measured here is called malondialdehyde which is gotten from the oxidation of polyunsaturated fatty acids. Its value in flaxseed oil was 5.39 ppm which is significantly low and can reflect acceptability in terms of quality. The hydrolytic state of the oil was evaluated by determining the acid value, so as to quantify the fatty acids released. In this study, the acid value of flaxseed oil was 2.82 mg KOH/g which is < 4 mg KOH/g, the standard acid value for crude and virgin vegetable oils as reported by FAO/WHO (2009). Lastly, the quantification of unsaturation in the oil done by determining the iodine value was 183.11 g *I*_2_/100 g characteristic of the presence of polyunsaturated fatty acids in significant proportion. According to previous reports, flaxseed oil contains about 87.8%–89.8% unsaturated fatty acids amongst which linoleic (26.2%) and α‐linolenic (42.5%) acids are the most abundant (Yacoob and Hassan 2016; Ishag et al. 2020).

Body weight is a sensitive and integrative indicator of overall health, nutritional status, and systemic response to disease and treatment (Raiten et al. 2025). Results showed that the normal control group exhibited a steady and progressive increase in body weight, which is consistent with healthy physiological growth in rodents under normal metabolic and dietary conditions (Ghasemi et al. 2021). This serves as a reliable baseline, indicating that neither malnutrition nor external stressors influenced the animals' growth curve. In contrast, the negative control group (DMBA alone) and the group taking flaxseed oil showed a marked reduction in weight gain, with body weights remaining relatively stagnant or declining slightly. This aligns with previous studies indicating that DMBA disrupts metabolic homeostasis, induces systemic oxidative stress, and can influence appetite and nutrient absorption effects commonly observed in cancer (Jovanović et al. 2023).

Changes in organ weights can be the consequence of toxicity, inflammation, and disease progression in animal models, especially in chemically induced carcinogenesis (Abd El‐Kader and Saiem Al‐Dahr 2016). Results showed no significant variation in the weight of all organs across groups except for the pancreas where the DMBA‐treated groups exhibited significantly lower pancreas weight. The significant reduction in pancreas weight can be attributed to pancreatic atrophy, potentially due to oxidative damage caused by the DMBA. This observation is in line with findings of El‐Missiry and El Gindy (2000), who reported that DMBA leads to oxidative damage in multiple organs, through oxidative stress and inflammation. Similar information was stated by Zingue et al. (2024) with some organs of ovariectomized rats with breast cancer treated with Njansang oil.

In the process of mammary cancer development, some changes in the lipid profile can be observed. These could include an increase in cholesterol (total), triglycerides, LDL cholesterol, but a reduction in HDL cholesterol. This dyslipidemia is generally the consequence of tumor development and the metabolic need for lipids and cholesterol in general. Additionally, it was reported that the chemotherapy of breast cancer can lead to important alterations of the lipid profile as mentioned above (Abdelsalam et al. 2012; Alimperti et al. 2023). Results revealed that the negative control group presented lower HDL levels compared to the groups taking DHA and flaxseed oil. It also exhibited the highest LDL level. This observation can be explained by the high aggressiveness of the cancer in the negative control group compared to the groups taking DHA and flaxseed oil (Abdelsalam et al. 2012). Higher HDL levels are generally associated with anti‐inflammatory effects and reduced cancer risk. HDL inhibits cytokines that boost tumor growth, support cholesterol outflow from cancer and immune system cells, and stop oxidative stress pathways that help delay tumor growth and control the tumor microenvironment (Yin et al. 2019). On the other hand, high LDL levels, as observed in the negative control group, promote tumor growth. This can be related to the absence of treatment in the negative control and especially the aggressiveness of the DMBA. The low LDL levels in groups taking DHA and flaxseed oil reflect their protective action in DMBA tumor development. The fatty acids involved (DHA and probably α‐linolenic acid) could be responsible for the observed effects. It has been reported that DHA has the ability to reduce the LDL cholesterol level within the lipid rafts of breast cancer cell membranes, which are capital for cell movement, invasion, and the whole cancer growth (Fabian et al. 2015). The high HDL and low LDL recorded in the group taking flaxseed oil can be attributed to the presence of polyunsaturated fatty acids, mostly omega‐3 fatty acids that are known for their beneficial roles on the heart (Cartolano et al. 2022). Reports show that docosahexaenoic and eicosapentaenoic acids modestly increase HDL levels mainly by altering lipid metabolism, reducing the amount of triglycerides, which protects HDL from degrading prematurely, and by remodeling HDL molecules to be bigger and more efficient at reverse cholesterol transport (Peña‐de‐la‐Sancha et al. 2023). On the other hand, α‐linolenic acid, which is an essential fatty acid from plant origin, was reported to modulate lipid profiles, mainly by reducing total cholesterol, LDL, and triglycerides, and slightly increasing HDL (Bertoni et al. 2023). It is, however, very important to note that α‐linolenic acid can be converted into DHA and EPA in vivo through desaturation and elongation in mammals. However, this conversion in humans is highly limited: < 8% for the conversion of α‐linolenic acid into eicosapentaenoic acid, and < 4% for the conversion of α‐linolenic acid into docosahexaenoic acid (Greupner et al. 2018). It is also important to note that α‐linolenic acid requires transformation into longer‐chain fatty acids such as DHA and EPA to be able to exert its biological function while DHA, for example, is directly incorporated (Cambiaggi et al. 2023).

Cancer development and progression can also lead to significant changes on organs integrity and function (Chinnappan et al. 2023; Olawale et al. 2021). In the same line, antigens directed against the cancer cells being developed will see their concentration increase in this process (Zingue et al. 2024). Results showed significantly higher creatinine, LDH, AST, ALT and tumor marker antigen C15‐3 concentration in the negative control group compared to the groups taking DHA and flaxseed oil respectively. This can be explained by the protective effect of DHA and flaxseed oil against DMBA aggressively. Olawale et al. (2021) showed that DMBA administration to rats lead to important increase in ALT and AST levels, indicating liver and heart injuries, while treatment with antioxidant‐rich agents helped reduce the alterations. This strengthen the hypothesis that increase in hepatocellular enzyme observed in DMBA cancer models are reproducible and can be changed via nutritional or pharmacologic mediations. This demonstrates the hepatotoxic effect of DMBA and the probable therapeutic attenuation. DHA and flaxseed oil were particularly operational in decreasing ALT, AST and LDH concentrations, testifying anti‐inflammatory and hepatoprotective effect (Calder 2020; Wang et al. 2020). Similar remarks were made by Zingue et al. (2024) with the serum enzyme levels of menopause‐like conditions rats with DMBA induced breast cancer after Njansang oil administration. The fact that the creatinine level was significantly reduced in the groups taking DHA and flaxseed oil compared to the negative control can be explained by their renoprotective action by limiting the damages caused by the DMBA. Creatinine level informs on the functionality of the kidney. High creatinine levels are generally associated with renal dysfunctioning or disease (Lin et al. 2023; Selby et al. 2019). Reports showed that omega‐3 supplementation can decreases the creatinine concentration in female rats with breast cancer induced using DMBA by reducing kidney alteration and inflammation caused by the carcinogenic substance (Santo et al. 2023). DHA is an omega‐3 and flaxseed oil is rich in α‐linolenic acid which is also an omega‐3 fatty acid. This can explain the renoprotective effect recorded with them. Similar protection was observed by Zingue et al. (2024) on the kidney or menopause‐like conditions rats with breast cancer induced using DMBA upon Njansang oil administration.

CA15‐3 is a protein measured through a blood test which is used in the diagnosis of breast cancer tumor. It is also used to monitor breast cancer treatment and a value below 30 U/mL is considered normal (Ryu et al. 2023). This marker was meaningfully higher in the negative control group when compared to the normal group and the groups taking DHA and flaxseed oil. Duffy (2006) demonstrated that a high CA15‐3 level is generally linked to breast cancer progression and systemic inflammatory responses. This shows the ability of DHA and flaxseed oil in inhibiting cancer development and reducing inflammation. DHA and α‐linolenic acid (in flaxseed oil) might be liable for the detected effects. Comparable results were obtained by Zingue et al. (2024) with Njansang oil (an oil rich in α‐linolenic acid) in menopause‐like conditions female wistar rats with mammary cancer induced using DMBA.

DMBA induced breast cancer generally can alter the microstructure of some organs. Specimen can present a modified mammary gland with hyperplastic, irregular cell lining at the level of the epithelium, disrupted membrane, cancer cells proliferation at the stroma, etc. Metastasis can affect other organs including the liver, kidney, etc., which will present significant structural changes compared to the normal tissues (Feng et al. 2015). Results revealed the occurrence of mucin in the ductal lumen with a highly granulated cytoplasm, discontinued ducts, outgrowth of malignant cells in the mammary gland (tumor) of the negative control group which were absent in the groups that received DHA and flaxseed oil. Still in the negative control group, leukocyte infiltrations and mesangial expansion with sclerotic glomeruli were recorded at the level of the kidney while these observations were absent in groups taking flaxseed oil and DHA. Results also reported that in the brain, DMBA caused the formation of hyperchromatic nuclei in the CA1 region of the hippocampus, reflecting neuronal necrosis in the negative control group which was not noticed in the groups taking DHA and flaxseed oil. The negative control group also exhibited hyperchromatic nuclei in the dentate gyrus which is neurological degeneration that was not found in the treated groups. Leukocytes' infiltration was registered in the liver of the negative control which is a sign of inflammation. However, they were absent in the group that received DHA and flaxseed oil. The islets of the pancreas were reduced in size in the negative control but slightly altered in the treated groups. These observations clearly show that DHA and flaxseed oil exert a good protective effect on organs against the toxicity of the DMBA. These can be explained by the nature of DHA which is an omega‐3 fatty acid and the presence of α‐linolenic acid present in flaxseed oil which belongs to the same class of fatty acids (omega‐3). These results are in line with the findings of Zingue et al. (2024) who made similar observations in the kidney, spleen, lung, liver, and uterus of rats with cancer induced with DMBA and treated with Njansang oil.

## 5 Conclusion

The goal of this work was to appraise the impact of flaxseed oil on breast cancer tumor marker, histopathological and biochemical markers of female rats with DMBA‐induced mammary cancer in contrast to DHA. Results showed that the flaxseed oil used is acceptable in term of quality and stability as its quality indexes fall within the accepted ranges. It has high polyunsaturated fatty acids content since it has a higher iodine value. Flaxseed oil and DHA significantly reduce the creatinine, LDH, AST, ALAT and CA15‐3 tumor marker levels in rats with DMBA‐induced breast cancer compared to those of the negative control group. Flaxseed and DHA increase the total protein in the serum of rats compared to those in which cancer was induced but was not treated. Flaxseed oil and DHA showed a protective action on the mammary gland, brain, pancreas, heart, liver, and kidney by preserving their microstructures from significant damages caused by the DMBA reagent as it was observed with these same organs in the negative control group. Flaxseed oil and DHA are potential ingredients that can be helpful in the management and prevention of breast cancer conditions. Further studies need to be carried out to evaluate the effect of flaxseed oil and DHA on oxidative stress markers, inflammatory cytokines of rats with DMBA‐induced breast cancer.

## Author Contributions

**Stephane Zingue:** conceptualization, investigation, writing – original draft, writing – review and editing, visualization, validation, methodology, software, formal analysis, resources, supervision, data curation. **Fabrice Tonfack Djikeng:** conceptualization, investigation, writing – original draft, writing – review and editing, visualization, validation, methodology, software, formal analysis, resources, supervision, data curation. **Achidi Aduni Ufuan:** conceptualization, investigation, writing – original draft, writing – review and editing, visualization, validation, methodology, software, formal analysis, resources, supervision, data curation. **Ines Sorelle Fotsing:** conceptualization, investigation, writing – original draft, writing – review and editing, visualization, validation, methodology, software, formal analysis, data curation, resources. **Hilda Nji Aza:** conceptualization, investigation, writing – original draft, writing – review and editing, visualization, validation, methodology, software, formal analysis, resources, data curation.

## Funding

The authors have nothing to report.

## Disclosure

*Author approval and responsibility statement*: All authors have read and approved the final version of the manuscript. Fabrice Tonfack Djikeng had full access to all of the data in this study and takes complete responsibility for the integrity of the data and the accuracy of the data analysis.

## Ethics Statement

Animals were treated according to the international standard guideline for animal use. An ethical clearance (reference number UB‐IACUC No. 02/2025) was obtained from the Institutional Animal Care and Use Committee, University of Buea.

## Conflicts of Interest

The authors declare no conflicts of interest.

## Data Availability

The data that support the findings of this study are available from the corresponding author upon reasonable request.
